# A quantitative study of brainstem projections from lamina I neurons in the cervical and lumbar enlargement of the rat

**DOI:** 10.1016/j.brainres.2009.10.041

**Published:** 2010-01-13

**Authors:** Erika Polgár, Lorna L. Wright, Andrew J. Todd

**Affiliations:** Neuroscience and Molecular Pharmacology, Faculty of Biomedical and Life Sciences, West Medical Building, University of Glasgow, Glasgow G12 8QQ, UK

**Keywords:** Caudal ventrolateral medulla, Lateral parabrachial area, Periaqueductal grey matter, Nucleus tractus solitarius, Dorsal reticular nucleus, Spinothalamic tract

## Abstract

Lamina I of the rat spinal cord contains neurons that project to various brain areas including thalamus, periaqueductal grey matter (PAG), lateral parabrachial area (LPb), caudal ventrolateral medulla and a region in dorsal medulla that includes the nucleus tractus solitarius and dorsal reticular nucleus. We have shown that spinothalamic lamina I neurons are infrequent in rat lumbar enlargement, where they constitute ∼ 5% of the estimated 400 projection neurons on each side of the L4 segment (Al-Khater and Todd, 2009). They are more numerous in cervical enlargement, but the total number of lamina I projection neurons in this region was not known. Here we have used paired injections of retrograde tracers into the brainstem to estimate the number of lamina I projection cells in the C7 segment. Our results suggest that there are ∼ 215 lamina I projection cells per side, and that spinothalamic cells therefore make up ∼ 42% of this population. The proportion of lamina I projection neurons labelled from PAG is higher in cervical than lumbar enlargement, while the proportion labelled from dorsal medulla is similar in the two regions. We also found that lamina I cells in L4 that project to the dorsal medulla are included in the population retrogradely labelled from LPb, thus confirming the estimate that there are around 400 lamina I projection cells in this segment.

## 1 Introduction

Lamina I of the dorsal horn (Rexed, 1952) is innervated by primary afferents that respond to noxious and/or thermal stimuli (Light and Perl, 1979; Sugiura et al., 1986), and contains many projection neurons that transmit this information to the brain (Todd, 2002; Willis and Coggeshall, 2004). Retrograde labelling studies in the rat have indicated that lamina I neurons project to several brain regions including the thalamus, periaqueductal grey matter (PAG), lateral parabrachial area (LPb) and various parts of the medulla (Menétrey et al., 1982, 1983; Cechetto et al., 1985; Hylden et al., 1989; Lima and Coimbra, 1988, 1989; Burstein et al., 1990; Lima et al., 1991; Esteves et al., 1993; Li et al., 1996, 1998; Marshall et al., 1996; Guan et al., 1998; Todd et al., 2000; Spike et al., 2003; Al-Khater et al., 2008; Al-Khater and Todd, 2009). Medullary termination sites include the nucleus tractus solitarius (NTS) (Menétrey and Basbaum, 1987; Menétrey and de Pommery, 1991; Raboisson et al., 1996), dorsal reticular nucleus (Lima, 1990; Almeida and Lima, 1997) and a region between the lateral reticular nucleus and spinal trigeminal nucleus that has been defined as the caudal ventrolateral medulla (CVLM) (Lima et al., 1991; Todd et al., 2000; Spike et al., 2003).

It has been shown that many lamina I neurons can be labelled from more than one brain region. For example, most of those in the mid-lumbar spinal cord that project to thalamus or PAG can also be retrogradely labelled from the LPb (Hylden et al., 1989; Spike et al., 2003; Al-Khater and Todd, 2009), and there is extensive overlap at this segmental level between the populations labelled from LPb and CVLM (Spike et al., 2003). Although the majority of retrogradely labelled cells are found contralateral to the injection site, indicating a predominantly crossed projection, some are found on the ipsilateral side. We have shown that when injections are made into both sides of the LPb or CVLM, most lamina I cells in L4 that are labelled from the ipsilateral side are also labelled from the corresponding site on the contralateral side, which suggests that the majority of lamina I cells have purely contralateral projections, while a smaller number project bilaterally (Spike et al., 2003).

Based on the results of quantitative studies in which tracers were injected into LPb, PAG and CVLM, we estimated that there are ∼ 400 lamina I projection neurons on each side in the L4 segment of the rat, and that these make up approximately 6% of the total neuronal population in this lamina (Spike et al., 2003; Al-Khater et al., 2008). However, this estimate did not take account of lamina I neurons that were labelled from the dorsal medulla. We have recently reported that spinothalamic neurons are very infrequent in lamina I of the rat lumbar enlargement, with only around 15–20 on each side in the L4 segment (Al-Khater et al., 2008; Al-Khater and Todd, 2009), amounting to less than 5% of the projection neurons in this lamina. However, lamina I spinothalamic cells were far more numerous in the cervical enlargement (∼ 90 cells/side in the C7 segment), although this region contained fewer lamina I spinoparabrachial cells. Since we did not know the total number of lamina I projection cells in C7 we were unable to determine the proportion that belonged to the spinothalamic tract.

The present study was carried out to address two unresolved issues concerning projections from lamina I to the brainstem: (1) Are cells labelled from the dorsal medulla (NTS and/or DRt) included in the population labelled from LPb in the L4 segment? (2) In the cervical enlargement are most or all of the cells that are labelled from PAG, CVLM or dorsal medulla included in the population labelled from LPb? Answering the first of these questions will allow us to assess the reliability of our estimate of the number of lamina I projection neurons in the mid-lumbar cord (Spike et al., 2003). The answer to the second question will enable us to provide a similar estimate for the cervical enlargement, and thus determine the proportion of projection cells that belong to the spinothalamic tract at this level.

## 2 Results

### 2.1 Injection sites

Quantitative results for retrograde labelling in lamina I were obtained from 10 experiments in which two tracers (Fluorogold and cholera toxin B subunit, CTb) were injected into different brain regions. Details of the injections are provided in Table 1. In all experiments, one injection was made into the left LPb, while the other was targetted on the PAG (experiments 1–3), the CVLM (experiments 4–6) or the dorsal medulla (NTS and DRt) (experiments 7–10) on the left side. In each case the rostral injection consisted of Fluorogold and the caudal one of CTb, since it has been reported that injections of Fluorogold can reduce the number of spinal neurons labelled by a second tracer injected into a more rostral site (Bice and Beal, 1997).

**Fig. 1:**
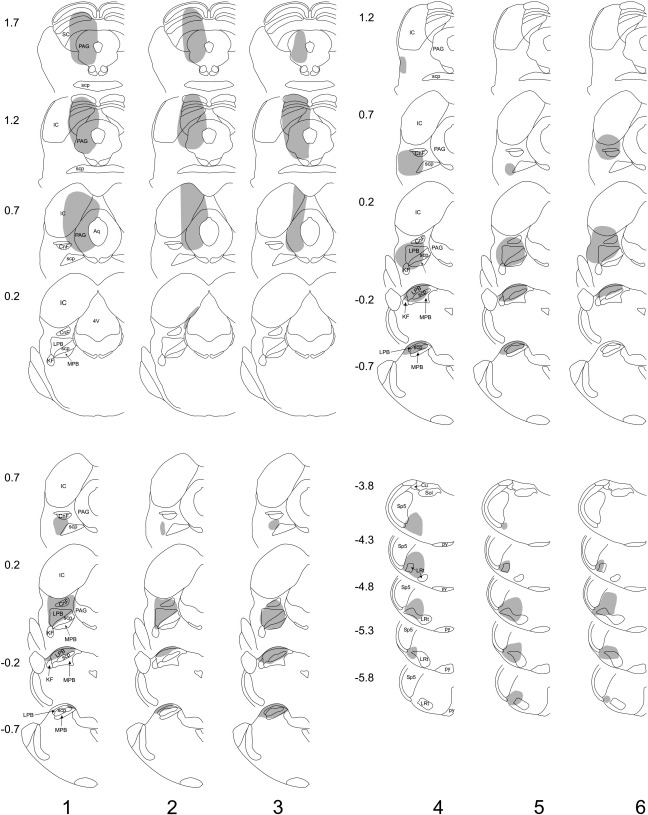
Injection sites in experiments 1–3 (Fluorogold injected into PAG, CTb into LPb) and 4–6 (Fluorogold injected into LPb, CTb into CVLM). Each vertical column represents a single experiment and the shaded area shows the spread of each tracer. Experiment numbers shown at the bottom correspond to those in Tables 1–2. The upper part of each column shows the rostral (Fluorogold) injection, and the lower part shows the caudal (CTb) injection. Numbers to the left of the drawings correspond to the position of the section anterior or posterior (−) to the interaural plane. Drawings are based on those of Paxinos and Watson (2005). 4V, 4th ventricle; Aq, aqueduct; CnF, cuneiform nucleus; Cu, cuneate nucleus; IC, inferior colliculus; KF, Kölliker–Fuse nucleus; LPB, lateral parabrachial area; LRt, lateral reticular nucleus; MPB, medial parabrachial area; PAG, periaqueductal grey matter; py, pyramid; SC, superior colliculus; scp, superior cerebellar peduncle; Sol, nucleus tractus solitarius; Sp5, spinal trigeminal nucleus.

**Fig. 2:**
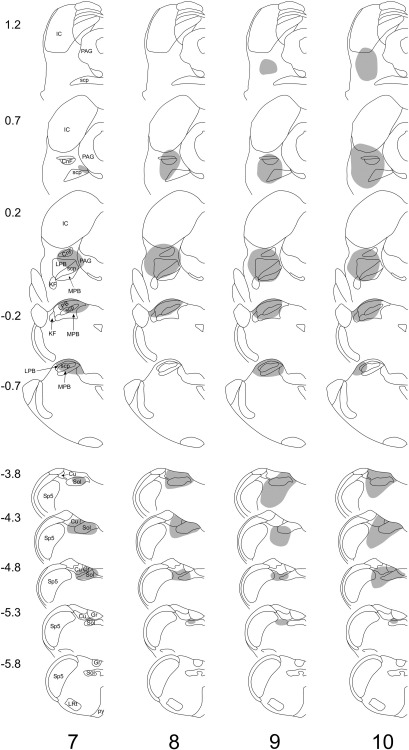
Injection sites in experiments 7–10 (Fluorogold injected into LPb, CTb into dorsal medulla). Each vertical column represents a single experiment and the shaded area shows the spread of each tracer. Experiment numbers shown at the bottom correspond to those in Tables 1–3. The upper part of each column shows the rostral (Fluorogold) injection, and the lower part shows the caudal (CTb) injection. Numbers to the left of the drawings correspond to the position of the section anterior or posterior (−) to the interaural plane. Drawings are based on those of Paxinos and Watson (2005). Gr, gracile nucleus; other abbreviations are as in Fig. 1.

**Fig. 3:**
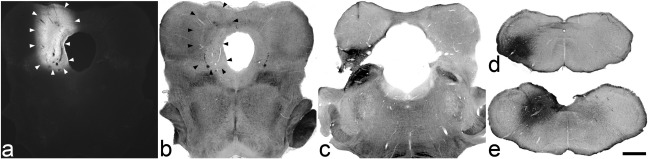
Photomicrographs of representative injection sites for each of the brainstem targets. Transverse sections through the brain are shown for injections into: (a, b) the PAG (experiment 2), (c) the LPb (experiment 1), (d) the CVLM (experiment 5), (e) the dorsal medulla (experiment 9). For the PAG injection, the spread of Fluorogold (outlined with arrowheads) is shown in a, which was taken with an UV filter set. The same field taken with bright-field optics is shown in b. (c–e) show CTb, which was revealed with an immunoperoxidase method. Scale bar = 1 mm.

Drawings of the spread of tracer are shown in Figs. 1 and 2, and representative photomicrographs through injection sites are illustrated in Fig. 3. Injections of Fluorogold into the PAG (experiments 1–3) were targetted on its caudal part and in each case these largely filled one side of the PAG at levels from ∼ 0.7 to 1.7 mm anterior to the interaural plane, without spread across the midline or into the LPb (Figs. 1, 3a,b). In each case there was also labelling within the superior and inferior colliculi. Injections of CTb (experiments 1–3) or Fluorogold (experiments 4–10) into the LPb filled most or all of this region, with variable spread of tracer into the medial parabrachial area, as well as the Kölliker–Fuse and cuneiform nuclei (Figs 1, 2, 3c). In some cases (experiments 1, 7 and 8), there was a very limited spread of tracer into the caudalmost part of the ventrolateral PAG at ∼ 0.2 mm anterior to the interaural plane. Injections of CTb targetted on the CVLM filled the lateral part of the lateral reticular nucleus between 4.3 and 4.8 mm posterior to the interaural plane and occupied the region between this nucleus and the spinal trigeminal nucleus (Figs. 1, 3d). CTb injections into the dorsal medulla occupied most or all of the NTS at ∼ 3.8 mm posterior to the interaural plane, with variable extension into this nucleus at more caudal levels. There was also some spread into the gracile and/or cuneate nuclei, as well as into the region in between NTS, spinal trigeminal and dorsal column nuclei, which has been defined as the dorsal reticular nucleus (Lima, 1990).

### 2.2 Retrograde labelling of lamina I neurons

**Fig. 4:**
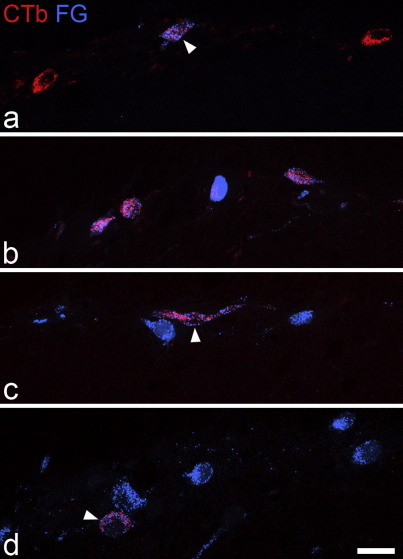
Examples of retrogradely labelled lamina I neurons from each type of experiment. All images show part of lamina I on the side contralateral to the injection, with CTb appearing red and Fluorogold (FG) blue. Panel a is from the C7 segment in experiment 3 (Fluorogold injected into PAG, CTb into LPb). This field shows 3 CTb-labelled cells, and one of these (arrowhead) also contains Fluorogold. Panel b is from the C7 segment in experiment 4 (Fluorogold injected into LPb, CTb into CVLM) and shows four retrogradely labelled cells, each of which contains both tracers. Panels c and d are from the C7 and L4 segments (respectively) of experiment 9 (Fluorogold injected into LPb, CTb into dorsal medulla). In both of these images there are cells that are labelled only with Fluorogold (which appear blue), as well as a single cell that is double-labelled (arrowhead). All images are projections of 5 optical sections at 2 μm z-spacing. Scale bar = 20 μm.

Quantitative analysis of retrogradely labelled lamina I neurons that contained one or both tracers was carried out on sections from the C7 segment in all experiments, and from the L4 segment in cases in which CTb had been injected into the dorsal medulla (Table 1). Results for the C7 segment in each experiment are shown in Table 2, while those for the L4 segment are in Table 3. In each case, the analysis was carried out on 10 randomly selected 60 μm thick Vibratome sections, and retrogradely labelled cells on the right side (contralateral to the injection site) were counted. Examples of retrogradely labelled lamina I neurons are shown in Fig. 4.

In experiments 1–3, in which Fluorogold was injected into the PAG and CTb into the LPb, between 40 and 60 cells that contained one or both tracers were identified in lamina I on the contralateral side in the 10 sections selected from C7. The great majority of these (85–100%) were labelled with CTb, while between 50% and 77% contained Fluorogold (Table 2).

In experiments 4–6 (Fluorogold injected into LPb, CTb into CVLM) the numbers of labelled lamina I cells in the 10 sections from C7 ranged from 54 to 79. Virtually all of these (98–100%) were labelled with Fluorogold, while between 83% and 89% were CTb-positive (Table 2).

In experiments 7–10 (Fluorogold injected into LPb, CTb into dorsal medulla) 40–60 labelled lamina I cells were present in the 10 sections from C7. Most of these (97–100%) were labelled with Fluorogold and 15–33% were labelled with CTb. In the L4 segment from these experiments between 70 and 113 lamina I neurons were labelled in the 10 selected sections. Most of these (96–98%) contained Fluorogold, while 22–27% were labelled with CTb.

## 3 Discussion

The main findings of this study were: (1) that in the C7 segment the great majority of lamina I neurons retrogradely labelled from PAG or CVLM were labelled from LPb, as we have previously reported for the mid-lumbar cord (Spike et al., 2003); and (2) that in both C7 and L4 segments most of the cells in this lamina that were labelled following injection of tracer into the dorsal medulla were also labelled from LPb.

### 3.1 Comparison with previous studies

Although it is possible that we underestimated the numbers of retrogradely labelled cells in these experiments due to lack of sensitivity for the detection of one or both tracers, we feel that this is unlikely for two reasons. Firstly, there was a clear distinction between cells that were positive and negative for each of the tracers, which suggests that none of the retrogradely labelled cells contained such low levels of tracer that these were close to the limits of detection. Secondly Al Ghamdi et al. (2009) have recently shown that following injection of CTb into the CVLM and Fluorogold into the LPb, immunostaining for the two tracers with a method identical to that used in the present study resulted in the detection of retrograde label in 99% of the large and medium-sized lamina I neurons (those with soma areas > 200 μm^2^ when viewed in the horizontal plane) that expressed the neurokinin 1 receptor, which is found on ∼ 80% of projection neurons in this lamina (Todd, 2002).

In two previous studies we have obtained estimates corresponding to 80 and 85 retrogradely labelled lamina I neurons per 600 μm in the L4 segment following injection of tracer into the contralateral LPb (Spike et al., 2003; Al-Khater and Todd, 2009). These estimates are in good agreement with the present result for the number of Fluorogold-labelled cells in the L4 segments of experiments 7–10 (mean of 87 cells per 600 μm).

Al-Khater and Todd (2009) estimated that the numbers of contralateral lamina I cells per 600 μm in C7 that were labelled from LPb and PAG were around 46 and 22, respectively. While the present result for LPb (53 cells/600 μm) is consistent with that, rather more cells labelled from PAG (32 cells/600 μm) were found in this study, and this can be attributed to a particularly high value in experiment 3. This discrepancy could have resulted from spread of Fluorogold into another region that is innervated by lamina I neurons. However, neither superior nor inferior colliculus (which were included in the Fluorogold injection site) receives a significant input from lamina I (Beitz, 1982; Menétrey et al., 1982; Bernard et al., 1995), and there was no extension of the injection into the LPb (Fig. 1). The most likely explanation for the larger number of spino-PAG cells in experiment 3 is therefore that it results from section to section variation in the number of retrogradely labelled cells.

Hylden et al. (1989) reported higher numbers of contralateral spinoparabrachial lamina I cells: 7.2 and 10.8 cells per 50 μm section in cervical and lumbar enlargements, respectively (corresponding to 86 cells/600 μm for cervical and 129 cells/600 μm for lumbar segments). However, they did not apparently correct for the over-counting that results from including transected cells at both section surfaces, and this probably accounts for the difference from our results.

The lower number of lamina I cells labelled from LPb in C7 compared to L4, which was also seen by Al-Khater and Todd (2009), is consistent with the results of Hylden et al. (1989). This difference is unlikely to be caused by a failure to detect significant numbers of spinoparabrachial neurons in the C7 segment, since the site of termination of spinal afferents to the LPb is similar for the two enlargements (Slugg and Light, 1994; Bernard et al., 1995; Feil and Herbert, 1995). In addition, we found that (apart from experiment 2) nearly all lamina I cells in C7 that were labelled from PAG, CVLM or dorsal medulla were also labelled from LPb, and this would not be expected if significant numbers of spinoparabrachial cells had not been detected.

The estimate for the number of lamina I cells in L4 that were retrogradely labelled from the contralateral dorsal medulla (22 cells/600 μm) is also consistent with that of 20 cells/700 μm in L3 that we reported previously following injections of CTb into this region (Todd et al., 2000). There was some variation between the injection sites within this series (experiments 7–10), particularly in the extent to which tracer entered the DRt. However, there was no clear relationship between the size and extent of the CTb injection and the number of retrogradely labelled cells in either C7 or L4. Although Lima (1990) identified the DRt as a major target for axons of lamina I neurons, Raboisson et al. (1996) reported that the main spinal inputs to this nucleus came from the deep dorsal horn. Small tracer injections that were largely restricted to the NTS have been shown to label significant numbers of lamina I cells (Menétrey and de Pommery, 1991) and ascending projections from superficial dorsal horn to NTS have been identified (Slugg and Light, 1994; Raboisson et al., 1996). Interestingly, in one experiment in which CTb was injected more laterally, resulting in extensive filling of DRt but with little labelling in the medial part of the NTS, we found very few lamina I cells labelled at either lumbar or cervical levels (A.J. Todd and E. Polgár, unpublished observations). It is therefore likely that the NTS, rather than the DRt, is the main dorsal medullary target for lamina I neurons, and the findings of Lima (1990) may be explained by spread of tracer into the NTS in her study.

### 3.2 The extent to which lamina I projection cells are labelled from LPb

Previous studies have provided evidence that most lamina I neurons in the lumbar enlargement that are retrogradely labelled from thalamus, PAG or CVLM can also be labelled from LPb. Spike et al. (2003) reported that following injection of tracers into PAG and LPb, 97% of lamina I spino-PAG cells in L4 were double-labelled, and Al-Khater and Todd (2009) found that 97% of lamina I spinothalamic cells in L3–5 segments were labelled from LPb. Spike et al. (2003) also observed that when injections were made into both LPb and CVLM, 85% of labelled cells contained Fluorogold transported from the LPb. More recently we have used the same injection strategy and found that a higher proportion (∼ 95%) were labelled from LPb (Al Ghamdi et al., in press). This difference was attributed to the increased sensitivity for detection of Cy5 (to reveal Fluorogold) of a gallium arsenide phosphide photomultiplier tube that was used in the latter study. The results of the present experiments extend these findings, by demonstrating that virtually all of the lamina I neurons labelled from the dorsal medulla are also included in the population labelled from LPb.

Al-Khater and Todd (2009) reported that 99% of spinothalamic lamina I neurons in C7 and C8 segments were retrogradely labelled from the LPb. The present results show that, as in the lumbar enlargement, most lamina I projection neurons in C7 can also be labelled by injection of tracer into the LPb, since in all but one of the experiments, 97–100% of the cells labelled from PAG, CVLM or dorsal medulla were double-labelled. In one experiment (#2), the proportion was lower (85%), but in this case the most medial part of LPb was not included in the CTb injection site and it is likely that this resulted in a reduced number of labelled cells.

Taken together, these findings suggest that the great majority (> 95%) of lamina I projection neurons in both lumbar and cervical enlargements can be retrogradely labelled by injections into the LPb. However, this does not necessarily mean that all of these cells belong to the spinoparabrachial tract, since some of the labelling may result from uptake of tracer by fibres passing through the injection site. For example, the projection from lamina I to the PAG passes through the rostral part of the parabrachial area (Bernard et al., 1995; Feil and Herbert, 1995), and although there is a dense terminal arborisation within the LPb it is possible that some axons pass through this region without contributing to this arborisation. If this is the case, then some spino-PAG neurons would not belong to the spinoparabrachial tract, but may be retrogradely labelled from the LPb. Spinothalamic axons from lamina I ascend near the parabrachial area and are located approximately 500 μm lateral to the external lateral nucleus of the LPb (J.F. Bernard, personal communication). Although these axons are likely to have been included in the LPb injections in several of the present series of experiments, this should not alter the interpretation, because our previous finding that virtually all spinothalamic lamina I neurons were labelled from LPb was obtained from cases in which the LPb injections did not extend into this region (Al-Khater and Todd, 2009). The uptake of tracer by fibres of passage is unlikely to have contributed to the labelling from the dorsal medulla, as these injections were located a considerable distance from the main ascending bundle of axons from lamina I, which is in the ventrolateral part of the brainstem at this level (Mehler, 1969; Zemlan et al., 1978; Slugg and Light, 1994). However, it causes a significant problem for interpreting the labelling that results from injections of tracer into the CVLM, as we have reported previously (Spike et al., 2003). Although tracer injections into CVLM cannot be used to identify supraspinal targets, they are useful because they can label a very high proportion of lamina I projection neurons in both enlargements.

### 3.3 Estimates of the number of lamina I projection neurons

Our previous estimate that there were ∼ 400 lamina I projection neurons on each side in the L4 segment of the rat was based on counts of cells retrogradely labelled from LPb, CVLM and PAG (Spike et al., 2003), and we have since demonstrated that all spinothalamic lamina I cells at this level are included in the population labelled from LPb (see above). Since nearly all lamina I neurons that project to the dorsal medulla are also labelled from LPb, this provides further support for the reliability of our estimate.

The present results, together with those of Al-Khater and Todd (2009) suggest that virtually all lamina I projection neurons in C7 can also be labelled from LPb. If the result from experiment 2 is excluded (because the medial part of LPb was not covered by this injection), the mean number of contralateral lamina I neurons labelled from LPb in C7 in the present study was 55.2/600 μm. The length of this segment is ∼ 2.3 mm (Al-Khater et al., 2008), and this is therefore equivalent to 212 cells in the entire segment. Since cells labelled from LPb made up between 98% and 100% of those labelled from other sites (with the exception of experiment 2) we estimate that there are ∼ 215 lamina I projection neurons on each side in C7.

If this interpretation is correct, the number of lamina I projection neurons is considerably lower in C7 than in L4, despite the similar size of the lamina in the two segments (Al-Khater and Todd, 2009). Another major difference is that a far higher proportion of these cells are included in the spinothalamic tract in C7: approximately 42% (90/215), compared to 5% for the L4 segment. The proportion that project to the PAG is also considerably higher in C7. Combining the present results with those from Al-Khater and Todd (2009) gives a mean of 27 contralateral lamina I spino-PAG neurons per 600 μm in C7, equivalent to 104 cells in the segment. These would therefore constitute 48% of lamina I projection neurons at this level, compared to ∼ 30% in L4 (Spike et al., 2003). In contrast, the proportion of lamina I projection neurons that are labelled from the dorsal medulla is similar at the two segmental levels: the estimated number in C7 is 49, corresponding to 23% of the projection cells, while that for L4 is 91 (assuming a segment length of 2.5 mm; Polgár et al., 2004), which is also 23%.

Although the smaller number of lamina I projection neurons in C7 compared to L4 is likely to reflect the much smaller size of its dermatome (Takahashi and Nakajima, 1996), it is not clear why there should be relatively more spinothalamic or spino-PAG neurons in the cervical enlargement. Information travelling from the dorsal horn to certain brain regions can arrive through more than one pathway, for example the amygdala receives inputs from both the LPb and the posterior triangular nucleus of the thalamus (Saper, 1995; Gauriau and Bernard, 2004). The larger number of spinoparabrachial cells in lumbar enlargement may therefore partially compensate for the reduced size of the spinothalamic tract at this level (Al-Khater and Todd, 2009).

## 4 Experimental procedures

### 4.1 Animals and operative procedures

All experiments were approved by the Ethical Review Process Applications Panel of the University of Glasgow and were performed in accordance with the European Community directive 86/609/EC and the UK Animals (Scientific Procedures) Act 1986. All efforts were made to minimise the number of animals used and their suffering.

Ten adult male Wistar rats (240–320 g; Harlan, Loughborough, UK) were anaesthetised with ketamine and xylazine (73.3 and 7.3 mg/kg i.p., respectively, supplemented as necessary) and placed in a stereotaxic frame. Each rat received two injections: (1) 50 nl of 4% Fluorogold (Fluorochrome Inc, Englewood, CO, USA) targetted on PAG or LPb of the left side, and (2) 200–250 nl of 1% cholera toxin B subunit (CTb; Sigma-Aldrich, Poole, UK) targetted on the left CVLM, LPb or dorsal medulla (NTS/DRt). The injection targets in each experiment are shown in Table 1. All injections were made through glass micropipettes, and in each case a different pipette was used for each tracer. The animals made an uneventful recovery from anaesthesia. After a 3-day survival period they were re-anaesthetised with pentobarbitone (300 mg i.p.) and perfused through the heart with a fixative that contained 4% freshly de-polymerised formaldehyde. The brain and lumbar spinal cord were dissected out and post-fixed for at least 4 h. The brain was cryoprotected in 30% sucrose overnight.

### 4.2 Tissue processing and immunocytochemistry

The regions of the brainstem that contained the injection sites were cut into 100 μm thick coronal sections with a freezing microtome. Sections through the Flurogold injection were mounted in anti-fade medium and viewed with epi-fluorescent illumination and an UV filter set. Sections through the CTb injection were reacted with goat anti-CTb (List Biological Laboratories, Campbell, CA, USA; diluted 1:50,000) by using an immunoperoxidase method as described previously (Todd et al., 2000). In all cases the spread of tracer from the injection sites was plotted onto drawings of the brainstem (Paxinos and Watson, 2005), and representative examples were photographed.

The C7 segments from all experiments as well as the L4 segments from experiments 7 to 10 (see Table 1), were notched on the left side (ipsilateral to the injections), to allow subsequent orientation, and were cut into 60 μm transverse sections with a Vibratome. These were incubated free-floating at 4 °C for 3 days in a cocktail consisting of guinea-pig anti-Fluorogold (Protos Biotech Corp., New York, USA, 1:500), goat anti-CTb (1:5000) and rabbit anti-NK1r (Sigma-Aldrich, 1:10,000). They were then reacted with species-specific secondary antibodies raised in donkey conjugated to either Alexa 488 (Invitrogen, Paisley, UK; 1:500), or to Rhodamine Red or Cy5 (Jackson Immunoresearch, West Grove, PA, USA; 1:100). The sections were mounted in anti-fade medium and stored at − 20 °C. The NK1r immunostaining was used to define the borders of lamina I (Todd et al., 1998).

### 4.3 Confocal microscopy and analysis

Transverse sections from the C7 segments of all 10 experiments, and from the L4 segments of experiments 7–10 were used to determine the numbers of retrogradely labelled lamina I neurons on the right (contralateral) side that contained one or both tracers. Ten sections were randomly selected and scanned sequentially (to avoid fluorescent bleed-through) through their full thickness with a confocal microscope (Bio-Rad Radiance 2100; Bio-Rad, Hemel Hempstead; UK), using 20 × dry and 40× oil-immersion lenses. Confocal image stacks were analysed with Neurolucida for Confocal software (MicroBrightField Inc., Colchester, VT, USA). Cells were judged to be in lamina I if they lay within the dense plexus of NK1r-immunoreactivity that occupies this lamina (Todd et al., 1998). Retrogradely labelled neurons were only included in the sample if their nucleus (identified as a filling defect) was entirely contained within the Vibratome section, or if part of the nucleus was present in the first optical section in the z-series (corresponding to the top of the Vibratome section). They were excluded if part of the nucleus was present in the last optical section (Spike et al., 2003; Al-Khater et al., 2008).

## Figures and Tables

**Table 1:** Injection sites.

Experiment	CTb injection site (volume nl)	Fluorogold injection site (volume nl)	Segments analysed
1	LPb (200)	PAG (50)	C7
2	LPb (200)	PAG (50)	C7
3	LPb (200)	PAG (50)	C7
4	CVLM (200)	LPb (50)	C7
5	CVLM (200)	LPb (50)	C7
6	CVLM (200)	LPb (50)	C7
7	NTS/DRt (250)	LPb (50)	C7, L4
8	NTS/DRt (250)	LPb (50)	C7, L4
9	NTS/DRt (250)	LPb (50)	C7, L4
10	NTS/DRt (250)	LPb (50)	C7, L4

**Table 2:** Quantitative results from C7.

Experiment	Injection sites	LPb	PAG	CVLM	NTS/DRt	Double-labelled neurons	Total neurons	% labelled from LPb
1	LPb/PAG	39	20	–	–	19	40	98
2	LPb/PAG	35	29	–	–	23	41	85
3	LPb/PAG	60	46	–	–	46	60	100
4	LPb/CVLM	78	–	70	–	69	79	99
5	LPb/CVLM	58	–	52	–	51	59	98
6	LPb/CVLM	54	–	45	–	45	54	100
7	LPb/NTS-DRt	54	–	–	18	17	55	98
8	LPb/NTS-DRt	58	–	–	12	10	60	97
9	LPb/NTS-DRt	40	–	–	6	6	40	100
10	LPb/NTS-DRt	56	–	–	15	13	58	97
Mean		53	32	56	13			

Counts of lamina I neurons in C7 that were labelled from one or both injection sites, together with the total number of labelled cells identified and the percentage of these that were labelled from LPb. In each case, cells were counted in 10 randomly selected 60 μm Vibratome sections.

**Table 3:** Quantitative results from L4.

Experiment	Injection sites	LPb	NTS/DRt	Double-labelled neurons	Total neurons	% labelled from LPb
7	LPb/NTS-DRt	67	18	15	70	96
8	LPb/NTS-DRt	76	18	16	78	97
9	LPb/NTS-DRt	92	21	19	94	98
10	LPb/NTS-DRt	111	30	28	113	98
Mean		87	22			

Counts of lamina I neurons in L4 that were labelled from one or both injection sites in experiments 7–10, together with the total number of labelled cells identified and the percentage of these that were labelled from LPb. In each case, cells were counted in 10 randomly selected 60 μm Vibratome sections.
